# Etiology of eating disorders: creation of a new model using standardised nursing language

**DOI:** 10.1192/j.eurpsy.2025.2404

**Published:** 2025-08-26

**Authors:** R. V. Díaz, M. D. P. Montero López, B. Gómez Esteban, S. Montserrat García Font

**Affiliations:** 1 Community Mental Health Center San Carlos (El Escorial), Hospital El Escorial, San Lorenzo del Escorial; 2 BIOLOGICAL SCIENCES, La Universidad Autónoma de Madrid; 3 CSM EL ESCORIAL; 4 CSM RIVAS-VACIAMADRID , Madrid, Spain

## Abstract

**Introduction:**

Eating disorders (EDS) have become in recent decades an important focus of interest for basic and clinical research due to the increase in prevalence and incidence observed and, therefore, the evident need to prevent and provide a therapeutic response to situations that affect important sectors of the population. Normal eating behavior and its pathological deviations can only be understood if they are studied under the biopsychosocial approach, since, attending only to the eating aspect, we are ignoring a series of determining factors in the development of this process. Within these factors, important changes have been observed in recent times in relation to the demographic profile, going from being a disease studied predominantly in women, to extending to men and non-binary people or with other sexual and/or gender options.

**Objectives:**

The aim of this study is to propose a new model for the representation of Eating Disorders (EDS) using nursing care languages, which allows us to approach the description of people affected by this pathology and their environment, implementing the perspective of gender, and that facilitates the realization of better diagnoses.

**Methods:**

A systematic bibliographic review on EDS was carried out using databases from the different areas involved. The information was ordered and summarized in tables and concept maps. Next, a comparison was made between the traditional EDS model, described using medical language, and the new model proposed by the nursing discipline, where all the information previously obtained is codified in languages proposed by the North American Nursing Diagnosis Association (NANDA) and the Nursing Development Foundation (FUDEN) in the manual on Standardized Nursing Knowledge (CENES).

**Results:**

The results obtained indicate that, after coding and organizing the existing information through nursing languages, the diagnostic view can be broadened by incorporating a greater number of variables to carry out a more complete approach to EDs. This would make it possible to propose a new conceptual model of these disorders, addressing them not only as a disease, but as a product of the failure of a healthy individualization that leads people who suffer from them to not carry out their self-care adequately, exerting on their person a act of anti-self care.

**Conclusions:**

The energetic representation of care, characteristic of nursing, could be useful in care practice, so that the description of the phenomenology of care for each individual could be established, and it would also provide a series of action algorithms that would favor its implementation for health prevention.

**Disclosure of Interest:**

None Declared

